# Selective Utilization of Polyguluronate by the Human Gut *Bacteroides* Species

**DOI:** 10.3390/md23090348

**Published:** 2025-08-29

**Authors:** Nuo Liu, Ming Li, Xiangting Yuan, Tianyu Fu, Youjing Lv, Qingsen Shang

**Affiliations:** 1 Key Laboratory of Marine Drugs of Ministry of Education, Shandong Key Laboratory of Glycoscience and Glycotherapeutics, School of Medicine and Pharmacy, Ocean University of China, Qingdao 266003, China; liunuo@stu.ouc.edu.cn (N.L.); liming12025@163.com (M.L.); yuanxiangting2025@163.com (X.Y.); futianyufty413@163.com (T.F.); lvyoujing1988@163.com (Y.L.); 2 Laboratory for Marine Drugs and Bioproducts, Qingdao Marine Science and Technology Center, Qingdao 266237, China; 3 Marine Biomedical Research Institute of Qingdao, Qingdao 266071, China

**Keywords:** polyguluronate, *Bacteroides* species, gut microbiota, utilization, fermentation

## Abstract

Human gut *Bacteroides* species play crucial roles in the metabolism of dietary polysaccharides. Polyguluronate (PG), a major component of alginate, has been widely used in the food and medical industries. However, how PG is utilized by human gut *Bacteroides* species has not been fully elucidated. Here, using a combination of culturomics, genomics, and state-of-the-art analytical techniques, we elucidated in detail the utilization profiles of PG by 17 different human gut *Bacteroides* species. Our results indicated that each *Bacteroides* species exhibited a unique capability for PG utilization. Among all species tested, *Bacteroides xylanisolvens* consumed the highest amount of PG and produced the greatest quantity of short-chain fatty acids, suggesting that it may be a keystone bacterium in PG utilization. Mass spectrometry showed that PG was degraded by *B. xylanisolvens* into a series of oligosaccharides. Genomic analyses confirmed that *B. xylanisolvens* possesses a large and divergent repertoire of carbohydrate-active enzymes. Moreover, genomic annotation identified two enzymes, PL17_2 and PL6_1, in *B. xylanisolvens* that are potentially responsible for PG degradation. Altogether, our study provides novel insights into PG utilization by human gut *Bacteroides* species, which has important implications for the development of carbohydrate-based drugs from marine resources.

## 1. Introduction

Polyguluronate (PG) is a marine polysaccharide composed of α-1,4-linked L-guluronate residues [1,2,3]. As a major component of alginate and a multifunctional macromolecule, PG is widely used in the food and biomedical industries [1,2,3]. For example, oral administration of PG sulfate has been shown to be effective in treating immunological liver injury [4]. In addition, PG-based nanoparticles have recently emerged as promising carriers for the targeted delivery of the antioxidant resveratrol [5]. Dietary intake of PG has also been found to alleviate dextran sulfate sodium-induced ulcerative colitis by modulating the composition of the gut microbiota [6].

PG is a large macromolecule and is therefore poorly absorbed following oral intake [7,8]. Moreover, owing to its unique chemical structure and physical properties, it cannot be digested by mammalian intestinal enzymes [7,8]. Therefore, after oral intake, PG reaches the distal colon largely intact, where it is metabolized by the gut microbiota [8,9]. Indeed, previous studies have shown that PG is a readily fermentable carbohydrate for the human gut microbiota [10,11,12]. In addition, each enterotype of the human gut microbiota has been shown to exhibit a unique capacity for PG fermentation [13].

However, although it is well-established that PG can be metabolized by the human gut microbiota, it should be noted that this complex microecosystem comprises trillions of diverse microorganisms [14,15]. Moreover, each species of gut bacteria possesses unique metabolic capabilities [16,17,18]. In fact, to date, how PG is utilized by specific microbes within the human gut has not been fully elucidated.

In the present study, using a combination of culturomics, genomics, and state-of-the-art analytical techniques, we aimed to elucidate in detail the utilization profiles of PG by 17 different human gut *Bacteroides* species. *Bacteroides* species were selected for this study for two main reasons. First, they are prominent members of the human gut microbiota [19,20]. Second, they are known to play crucial roles in the metabolism of dietary polysaccharides [21,22,23]. By elucidating the complex interactions between PG and human gut *Bacteroides* species, we anticipate that our study will contribute to understanding both the metabolism and the therapeutic effects of this multifunctional macromolecule.

## 2. Results

### 2.1. Each Bacteroides Species Was Characterized with a Unique Capability for PG Utilization

**Figure 1:**
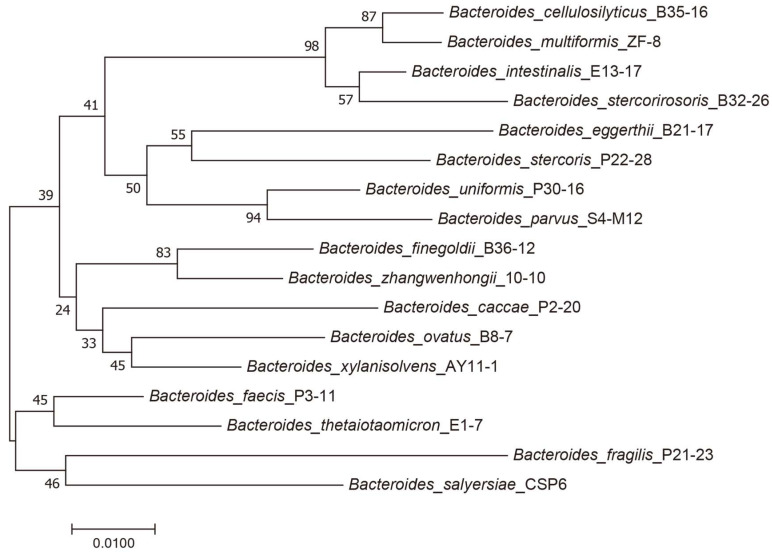
Phylogenetic tree of the 17 *Bacteroides* species included in this study.

A total of 17 taxonomically distinct *Bacteroides* species from the human gut microbiota were used in our study (Figure 1). These bacteria included *B. cellulosilyticus*, *B. multiformis*, *B. intestinalis*, *B. stercorirosoris*, *B. eggerthii*, *B. stercoris*, *B. uniformis*, *B. parvus*, *B. finegoldii*, *B. zhangwenhongii*, *B. caccae*, *B. ovatus*, *B. xylanisolvens*, *B. faecis*, *B. thetaiotaomicron*, *B. fragilis*, and *B. salyersiae*. All strains were originally isolated from fecal samples of healthy individuals and subsequently maintained in our laboratory collection [24,25]. All bacterial strains were cryopreserved in glycerol and stored at −80 °C. These strains are available from the corresponding author upon reasonable request.

**Figure 2:**
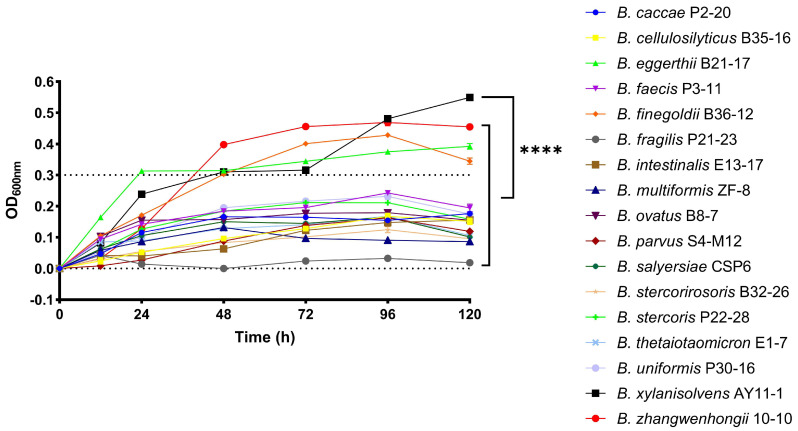
Growth curves of 17 phylogenetically distinct human gut *Bacteroides* species on PG. The OD at the wavelength of 600 nm was monitored from 0 h to 120 h. The fermentation experiment was performed in quadruplicate (*n* = 4). **** *p* < 0.0001. The error bars have been included in the growth curves; however, due to the very low variability between biological replicates, they are not easily distinguishable. In most cases the error bars are smaller than the symbols on the curve.

All bacteria were cultured anaerobically in a medium containing PG as the sole carbon source. Optical density (OD) at 600 nm was monitored, and bacterial growth curves were generated. We found that the growth curves differed markedly among the species (Figure 2). For example, *B. xylanisolvens* reached the highest OD, whereas *B. fragilis* reached the lowest. Furthermore, not all tested bacteria were capable of efficient growth (OD ≥ 0.3) in the PG-containing medium (Figure 2). Specifically, only four *Bacteroides* species—*B. xylanisolvens*, *B. zhangwenhongii*, *B. eggerthii*, and *B. finegoldii*—showed active growth under these conditions (Figure 2). Given that PG served as the only carbon source in the medium, these results indicate that each *Bacteroides* species exhibited a unique capability for PG utilization.

### 2.2. B. xylanisolvens Was Potentially a Keystone Bacterium Responsible for PG Utilization

Given that *B. xylanisolvens* exhibited the highest OD in the medium containing PG as the sole carbon source (Figure 2), we hypothesized that this species might be a keystone bacterium in PG utilization. To test this, we compared PG consumption among the *Bacteroides* species during fermentation.

**Figure 3:**
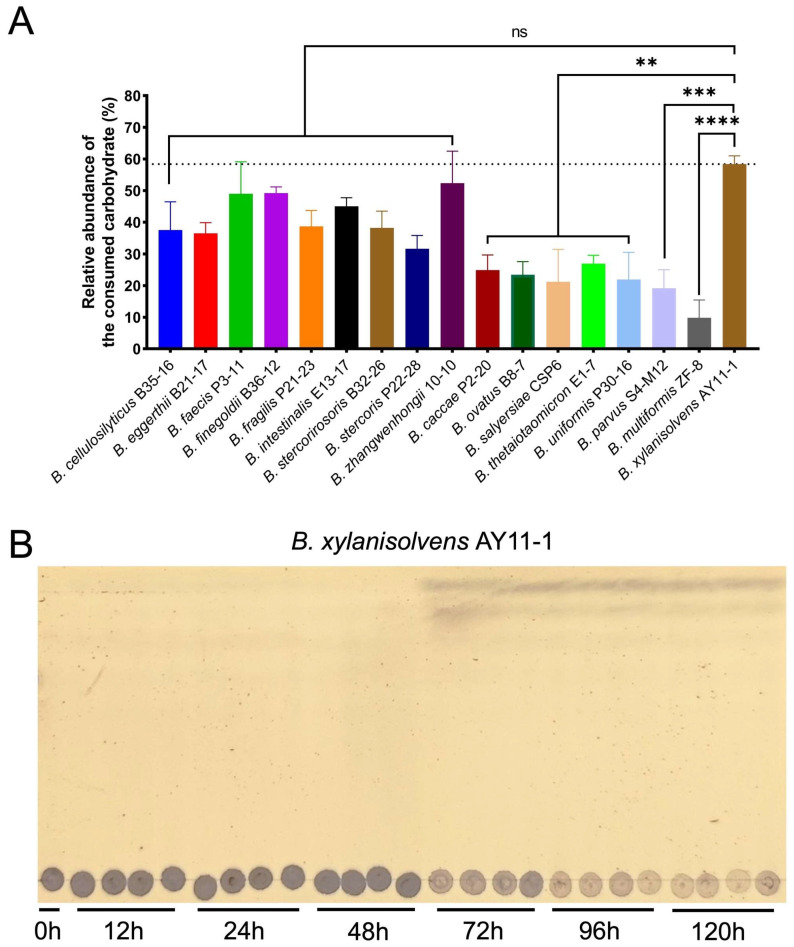
Utilization of PG by 17 phylogenetically distinct human gut *Bacteroides* species. Relative amount of PG consumed by each species (**A**). TLC analysis of PG utilization by *B. xylanisolvens* AY11-1 (**B**). The fermentation experiment was performed in quadruplicate (*n* = 4). ** *p* < 0.01; *** *p* < 0.001; **** *p* < 0.0001; ns, not significant.

Interestingly, among all the tested bacterial strains, *B. xylanisolvens* exhibited the most pronounced capacity for PG consumption, as quantitatively demonstrated by the marked reduction in PG substrate in the culture medium (Figure 3A). This superior utilization capability was further corroborated by thin-layer chromatography (TLC) analysis, which revealed a substantial degradation of PG in samples inoculated with *B. xylanisolvens* (Figure 3B).

Collectively, these findings highlight *B. xylanisolvens* as an efficient degrader for PG, implicating its potential ecological role in the utilization of this marine polysaccharide within the human gut microbiota.

Short-chain fatty acids (SCFAs) are a group of critically important organic compounds produced by the human gut microbiota during carbohydrate fermentation [26,27,28]. In this regard, we next analyzed the production of SCFAs by the human gut *Bacteroides* species.

**Figure 4:**
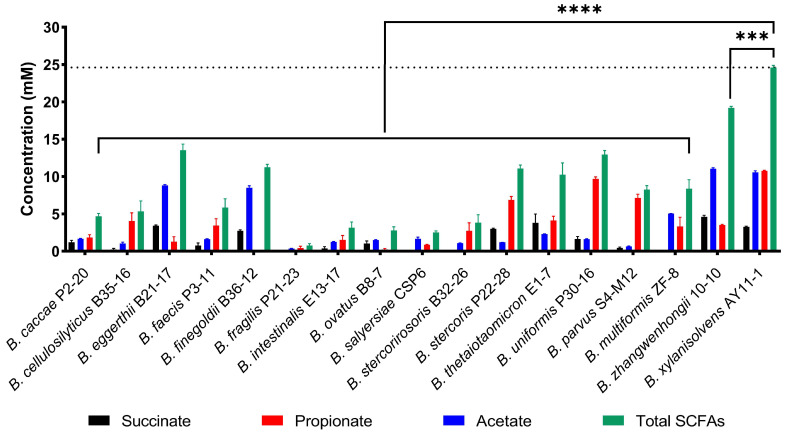
Production of SCFAs by 17 phylogenetically distinct *Bacteroides* species from the human gut microbiota. The fermentation experiment was performed in quadruplicate (*n* = 4). *** *p* < 0.001; **** *p* < 0.0001.

High-performance liquid chromatography (HPLC) analysis showed that the SCFAs produced were primarily succinate, propionate, and acetate (Figure 4). Notably, among all tested bacteria, *B. xylanisolvens* produced the highest amount of SCFAs during PG fermentation (Figure 4). Combined with the observations that *B. xylanisolvens* had grown to the highest OD and consumed the largest quantity of PG, these results collectively indicate that *B. xylanisolvens* is a keystone bacterium responsible for PG utilization.

### 2.3. PG Was Degraded into a Series of Oligosaccharides by B. xylanisolvens

We next sought to explore how PG was utilized by the candidate keystone bacterium *B. xylanisolvens*. To address this, we analyzed the degradation products of PG using mass spectrometry (MS).

**Figure 5:**
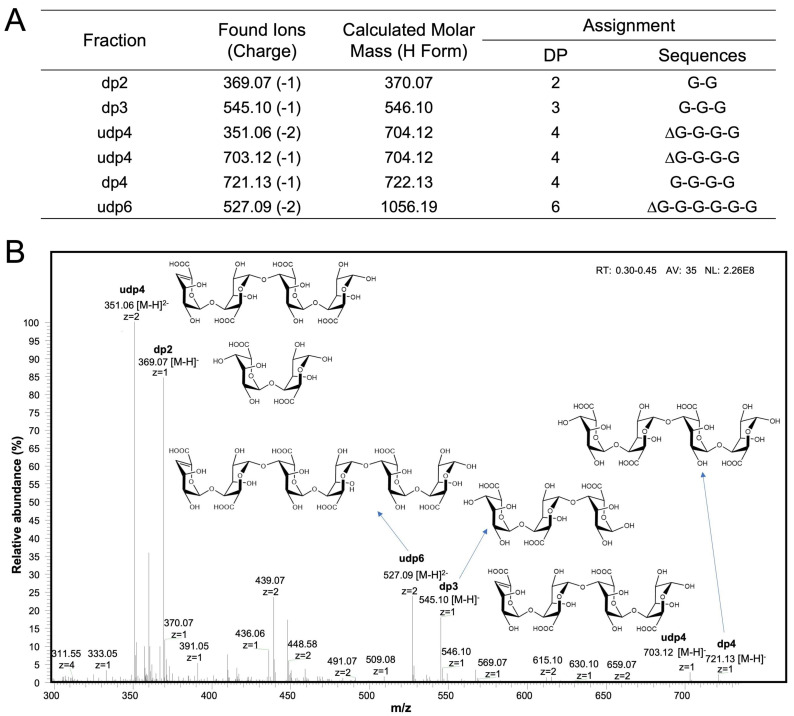
MS analysis of PG degradation products by *B. xylanisolvens*. Oligosaccharides produced during fermentation are shown. Assignment of detected ions (**A**). Chemical structures of the PG-derived oligosaccharides (**B**).

Interestingly, we found that PG was degraded into a series of oligosaccharides with degrees of polymerization (dp) ranging from 2 to 6 (Figure 5A). These oligosaccharides consisted of both saturated and unsaturated forms (Figure 5B). Specifically, the saturated oligosaccharides included disaccharide (dp2), trisaccharide (dp3), and tetrasaccharide (dp4), while the unsaturated oligosaccharides were identified as unsaturated tetrasaccharide (udp4) and unsaturated hexasaccharide (udpP6) (Figure 5B).

We also analyzed the production of PG oligosaccharides by other bacteria (Figures S1–S3). Similarly, *B. eggerthii*, *B. finegoldii*, and *B. zhangwenhongii* were also capable of producing PG oligosaccharides, although they reached a lower OD and consumed less carbohydrate compared to *B. xylanisolvens* (Figures S1–S3). Together, these findings suggest that PG is likely initially degraded into oligomers before being utilized and fermented by human gut *Bacteroides* species.

### 2.4. Genomic Analysis Linked PL17_2 and PL6_1 to PG Degradation in B. xylanisolvens

**Figure 6:**
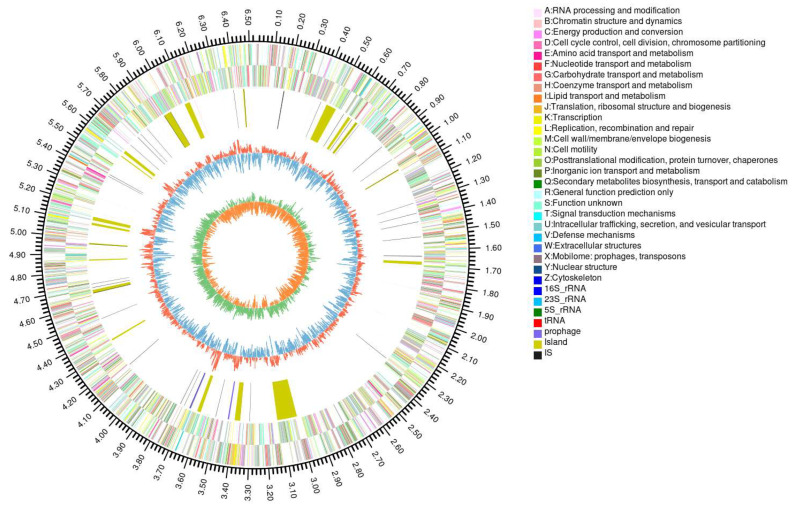
Analysis of the genome of *B. xylanisolvens* AY11-1. Circos plot shows the genomic composition of the bacterium.

To investigate how PG oligosaccharides are produced by human gut *Bacteroides* species, we sequenced the genome of the candidate keystone bacterium *B. xylanisolvens*. The genome was determined to be 6,530,506 bp in length with a GC content of 42.12%, and no plasmids were detected (Figure 6).

**Figure 7:**
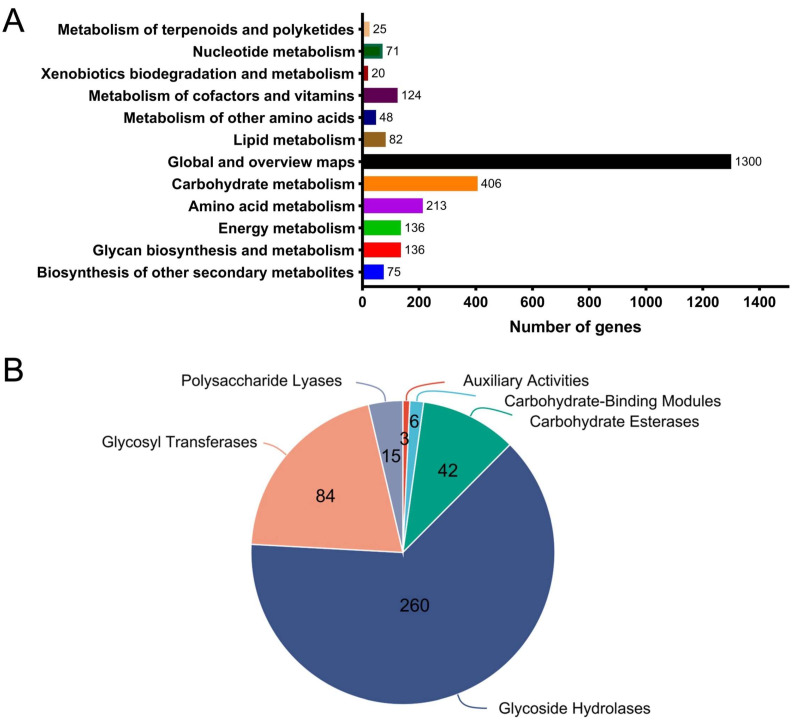
Genomic and CAZyme analysis of *B. xylanisolvens* AY11-1. KEGG pathway annotation (**A**). CAZyme family classification (**B**).

Kyoto Encyclopedia of Genes and Genomes (KEGG) pathway analysis revealed that 406 genes are involved in carbohydrate metabolism and that 136 genes are associated with glycan biosynthesis and metabolism (Figure 7A), suggesting that *B. xylanisolvens* is highly adept at degrading and metabolizing complex dietary carbohydrates.

The gut microbial carbohydrate active enzymes (CAZymes) have been documented to play crucial roles in the metabolism of dietary polysaccharides [29,30]. Specifically, these enzymes could break down complex carbohydrates into smaller and absorbable components (monosaccharide, disaccharide, and trisaccharide, etc.) [31,32]. Therefore, we analyzed the CAZyme repertoire in the genome of *B. xylanisolvens*.

Interestingly, a total of 410 genes were annotated as encoding CAZymes in the genome of *B. xylanisolvens* (Figure 7B). In addition, all six classes of CAZymes including polysaccharide lyases (PLs), glycoside hydrolases (GHs), auxiliary activities (AAs), glycosyltransferases (GTs), carbohydrate-binding modules (CBMs), and carbohydrate esterases (CEs) were all identified the genome of *B. xylanisolvens* (Figure 7B). Consistent with previous studies [24,33], these results reinforce that *B. xylanisolvens* is a proficient polysaccharide-degrading bacterium within the human gut microbiota.

Previous studies have well demonstrated that alginate lyases and oligo-alginate lyases are two classes of polysaccharide lyases (PLs) that catalyze the cleavage of glycosidic bonds in alginate, PG, and polymannuronate (PM) [34,35]. In our study, the production of unsaturated PG oligosaccharides by *B. xylanisolvens* (Figure 5) implied the involvement of specific PLs in PG utilization. Therefore, we next analyzed all PLs encoded in the genome of *B. xylanisolvens*.

Interrogation of the Polysaccharide-Utilization Loci DataBase (PULDB) revealed a candidate locus, PUL52 (https://www.cazy.org/PULDB/index.php?pul=73618, accessed on 25 August 2025), which is predicted to be involved in the degradation of alginate-like substrates in *B. xylanisolvens*. This PUL encodes a suite of proteins typical for polysaccharide utilization, including SusC- and SusD-like homologs for substrate binding and transport, a protein from family PL17_2, and a protein from family PL6_1. Given that both PL6 and PL17 families are known to contain alginate lyases active on PG [34,35], we propose that PUL52 is the primary genetic locus enabling *B. xylanisolvens* AY11-1 to degrade and utilize PG. The oligosaccharides generated by the action of these lyases are likely imported into the cell for further metabolism.

## 3. Discussion

### 3.1. Strengths and Key Findings of the Study

Accumulating evidence has indicated that *Bacteroides* species are critically important taxa of the human gut microbiota [19]. They play fundamental roles in maintaining intestinal eubiosis [20] and make substantial contributions to the metabolism of undigested dietary polysaccharides [21,22,23]. This study focused on elucidating the complex interactions between human gut *Bacteroides* species and the marine polysaccharide PG.

Using an integrated approach combining culturomics, genomics, and advanced analytical techniques, we provide new insights into PG utilization by the human gut *Bacteroides* species, particularly *B. xylanisolvens*. Our results indicated that each *Bacteroides* species exhibited a unique capability for PG utilization. Furthermore, we discovered for the first time that *B. xylanisolvens* likely serves as a keystone bacterium in PG utilization. We also demonstrated that *B. xylanisolvens* degrades PG into a series of oligosaccharides, and we identified PL17_2 and PL6_1 in this strain, which are potentially responsible for PG degradation.

Our findings provide critical insights that can inform the development of PG-based therapeutic strategies. The premise of using PG as a delivery vehicle or a drug relies on its ability to reach specific segments of the gastrointestinal tract intact before being degraded and utilized. Our study demonstrates that while PG is susceptible to degradation by certain bacteria in the human gut, this activity is not universal but is instead a specialized function primarily found in specific species like *B. xylanisolvens*. This variability suggests that the in vivo stability and therapeutic window of a PG-based drug could be highly dependent on an individual’s gut microbiome composition. Therefore, the development of PG-based therapeutics should account for inter-individual variations in the intestinal microbiome to achieve consistent and predictable efficacy. However, the challenge of microbiome variability in drug delivery is now being addressed through emerging strategies such as the GlycoCaging system [36]. This approach employs bespoke plant glycoconjugates to enable selective drug activation by specific bacterial glycosidases in the human colon, thereby minimizing systemic exposure and enhancing localized efficacy. Our findings suggest that similar principles could be adopted to improve PG-based therapeutic systems, leveraging enzyme-specific activation to overcome individual variations in gut microbiota composition.

### 3.2. Outlooks and Future Directions in the Field

In the current study, we illustrated that PG is degraded into oligomers by the human gut *Bacteroides* species. It is anticipated that these oligomers would be produced in the human intestine following oral consumption of PG. However, the effects of these oligomers on intestinal microecology and their potential for intestinal absorption remain unexplored, representing a key direction for future research.

Inter-individual variation in the capacity to degrade dietary glycans is increasingly recognized as a key factor influencing both host metabolism and gut microbiota composition [37,38]. Results from the present study suggest that dietary exposure to specific polysaccharides, including the algal-derived substances like PG, may select specialized degraders such as *B. xylanisolvens*. *B. xylanisolvens* has been detected in the human gut microbiomes across the globe but with inconsistent prevalence rates [39]. Further large-scale, multi-ethnic cohort studies are therefore warranted to explicitly link geographic, genetic, and dietary factors with functional potential for PG degradation in the human gut microbiome.

*B. xylanisolvens* has long been proposed as a next-generation probiotic bacterium [24,40,41,42]. Our study shows that it utilizes PG efficiently, opening new avenues for investigating the pharmacological effects of PG through the lens of *B. xylanisolvens* metabolism. This intriguing connection warrants more detailed investigation to elucidate the underlying mechanisms and potential applications.

The divergent growth patterns of *Bacteroides* species on PG align with known genetic variations in the polysaccharide utilization loci (PULs) among different bacteria in the human gut [43,44,45]. Although PG and alginate were once considered indigestible, recent studies reveal that only certain species possess specialized enzymes for its degradation [8,10,24]. Our results further demonstrate that even among these, significant differences exist in PG metabolism, with *B. xylanisolvens* exhibiting superior growth—possibly due to a more efficient PG-degrading apparatus. These findings underscore the functional niche specialization within human gut microbes, likely driven by genomic capacity for specific dietary glycans. Future comparative omics studies are warranted to elucidate the key enzymes and regulators underlying these phenotypic differences.

The genome of *B. xylanisolvens*, which is known to be richly endowed with CAZymes as reported in previous studies [33,46], was found in our analysis to encode a total of 410 CAZymes. These findings emphasize the need for future functional proteomic studies to express and characterize the enzymes encoded by these candidate genes to definitively map the biochemical pathway of PG degradation in the human gut microbiota.

The distinct oligosaccharide profiles generated by efficient degraders like *B. xylanisolvens* suggest that PG hydrolysis could initiate a cross-feeding network within the human gut ecosystem. In such a scenario, primary degraders break down complex dietary polysaccharides into smaller oligosaccharides, which may then be utilized as nutrients by other community members that lack the initial degradative machinery [47,48]. This metabolic cooperation is a fundamental driver of microbial diversity and stability in the gut [47,48]. The fact that only a subset of species possesses the ability to utilize PG efficiently positions them as potential keystone species that facilitate the sharing of public goods as recently highlighted in the context of other dietary glycans such as chondroitin sulfate and hyaluronic acid [25,49]. Future studies co-culturing efficient PG degraders with non-degraders will be essential to validate the existence and extent of such cross-feeding interactions.

### 3.3. Study Limitations

The starch utilization system (Sus) in human gut *Bacteroides* species has been documented to play critical roles in dietary polysaccharide metabolism [43,44,45]. In the present study, however, the specific Sus proteins involved in PG metabolism by *B. xylanisolvens* remain to be fully characterized. Additionally, although we identified PL17_2 and PL6_1 in the genome of *B. xylanisolvens*, their enzymatic activities have yet to be confirmed through biochemical and genetic approaches. Elucidating the molecular mechanism via genetic manipulation remains a primary goal for our future research. Finally, as keystone taxa of the human intestinal microbiome [19,20], more than 50 *Bacteroides* species have been reported to date [19,20]. Our current study included only 17 of these species; thus, more comprehensive investigations are warranted to generalize these findings across a broader taxonomic range.

## 4. Materials and Methods

### 4.1. Chemicals and Reagents

PG was prepared from the brown alga *Laminaria japonica* using the methods described elsewhere [6]. The molecular weight (Mw) of PG was determined to be 8.59 kDa [6]. Compositional analysis of PG using nuclear magnetic resonance (NMR) spectroscopy in our previous study indicated a molar ratio of 91.86% *L*-guluronate to 8.14% *D*-mannuronate, confirming that the PG preparation was a nearly homopolymer of *L*-guluronic acid [6]. The VI medium was used for the in vitro anaerobic fermentation experiments. PG was added to the VI medium as the sole carbon source at a concentration of 8 g/L following the previous protocol [13,24]. The standard SCFAs solutions were acquired from Sigma-Aldrich (St. Louis, MO, USA).

All other reagents and chemicals used for the preparation of VI medium were of analytical grade. The hemin and L-cysteine hydrochloride were obtained from Sangon Biotech (Shanghai, China). The nitrogen source including tryptone, peptone, and yeast extract were all purchased from Sigma-Aldrich (St. Louis, MO, USA). The guluronic acid was obtained from Qingdao Haida Marine Oligose Technology (Qingdao, China).

### 4.2. Bacterial Strains

In the present study, we collected a total of 17 phylogenetically distinct *Bacteroides* species from the human gut microbiota. These bacteria included *B. cellulosilyticus* B35-16, *B. multiformis* ZF-8, *B. intestinalis* E13-17, *B. stercorirosoris* B32-26, *B. eggerthii* B21-17, *B. stercoris* P22-28, *B. uniformis* P30-16, *B. parvus* S4-M12, *B. finegoldii* B36-12, *B. zhangwenhongii* 10-10, *B. caccae* P2-20, *B. ovatus* B8-7, *B. xylanisolvens* AY11-1, *B. faecis* P3-11, *B. thetaiotaomicron* E1-7, *B. fragilis* P21-23, and *B. salyersiae* CSP6. All the bacteria have been previously isolated from the fresh fecal samples of healthy individuals. Some of the bacterial strains have been previously reported in our work [24,25].

A phylogenetic tree of the human gut *Bacteroides* species was constructed based on the 16S rDNA gene sequences of the bacteria (Table S1). The analysis was performed using the Molecular Evolutionary Genetics Analysis (MEGA) software (version 7.0.26) as preciously described [24,25].

### 4.3. In Vitro Fermentation

All the bacteria were inoculated into the VI medium containing PG as the sole carbon source. The fermentation experiments were carried out at 37 °C in an anaerobic chamber. The chamber (product model, AW 500SG) was obtained from Electrotek (Shipley, West Yorkshire, UK). The gases in the chamber consisted of 80% N_2_, 10% H_2_, and 10% CO_2_. The OD at 600 nm of the culture medium was monitored from 0 h to 120 h using the ReadMax 1200 microplate spectrophotometer. The instrument was obtained from Shanghai Flash Spectrum Biological Technology (Shanghai, China). The fermentation experiment was performed in quadruplicate (*n* = 4).

### 4.4. Carbohydrate Utilization Analysis

The concentration of PG in the VI medium was analyzed using the phenol-sulfuric acid method [13,25,50,51]. Guluronic acid was used as a standard for the analysis as it was the building block of PG. By reacting with phenol, the breakdown products of PG produced a yellow-gold color. The absorbance of the resulting solution was measured spectrophotometrically using the aforementioned ReadMax 1200 microplate spectrophotometer. The wavelength of the spectrophotometer was set at 490 nm for the analysis.

The TLC analysis was performed to check the utilization of PG by the candidate keystone bacterium *B. xylanisolvens*. Briefly, the culture medium was collected at different time points. After that, the medium was filtered using a MF-Millipore 0.22 µm membrane from Merck KGaA (Darmstadt, Hessen, Germany). Then, about 0.6 μL aliquot of the resulting medium was loaded onto a pre-coated silica gel-60 aluminum plate from Merck KGaA (Darmstadt, Hessen, Germany). PG and its degraded oligomers were resolved using the formic acid/n-butanol/water (6:4:1, vol/vol/vol) solution as an eluent. The orcinol-sulfuric acid reagent was prepared as previously described and was used to visualize the carbohydrate on the plate [13,25].

### 4.5. SCFAs Analysis

The 1260 Infinity I high-performance liquid chromatography (HPLC) system from Agilent Technologies (Santa Clara, CA, USA) was used for the analysis of the SCFAs in the VI culture medium. The Aminex HPX-87H ion-exclusion column from Bio-Rad Laboratories (Hercules, CA, USA) was used for the analysis. The wavelength of the ultraviolet (UV) detector in the HPLC system was set at 210 nm as previously described [13,24,25]. The system was running isocratically using a 5.0 mM sulfuric acid solution as mobile phase.

### 4.6. Degradation Products Analysis

The PG oligosaccharides were purified from the culture medium using the PD MiniTrap G-10 gravity flow columns from Cytiva (Marlborough, MA, USA). The obtained oligosaccharides were dissolved in purified acetonitrile from Merck KGaA (Darmstadt, Hessen, Germany) and analyzed using the LTQ Orbitrap XL mass spectrometer from Thermo Fisher Scientific (Waltham, MA, USA). The mass spectrum data were acquired under the negative ion mode.

### 4.7. Genome Sequencing and Bioinformatics Analysis

The whole genome of the gut bacterium *B. xylanisolvens* AY11-1 was sequenced using both the Illumina HiSeq platform (San Diego, CA, USA) and the Oxford Nanopore Technologies (ONT) Nanopore PromethION platform (Oxford, Cambridge, UK). The sequencing experiments were conducted with the help from Majorbio Bio-Pharm Biotechnology (Shanghai, China). Bioinformatics analyses of the genomic sequencing data, including CAZymes annotation and KEGG pathway analysis, were performed using computational tools from the Majorbio Cloud Platform (www.majorbio.com, accessed on 30 June 2025) following the protocol previously described [25]. All six classes of the CAZymes including PLs, GHs, AAs, GTs, CBMs, and CEs were identified in accordance with established protocols and guidelines from the carbohydrate-active enzymes database (http://www.cazy.org/, accessed on 30 June 2025) [52,53,54,55,56,57]. The whole genome sequence of *B. xylanisolvens* AY11-1 was deposited in the GenBank under the accession number CP120351.1. The BioProject and BioSample accession numbers were PRJNA943352 and SAMN33717058, respectively.

### 4.8. Statistical Analyses

Data were expressed as mean ± standard error of mean (SEM). Statistical analyses were performed using one-way ANOVA (analysis of variance) with post hoc Tukey’s tests from GraphPad Prism (version 8.0.2) (San Diego, CA, USA). All results were considered statistically significant at *p* < 0.05. * *p* < 0.05; ** *p* < 0.01; *** *p* < 0.001; **** *p* < 0.0001.

## 5. Conclusions

Each *Bacteroides* species from the human gut microbiota was characterized with a unique capability for PG utilization. Among all species tested, *B. xylanisolvens* consumed the highest quantity of PG and produced the maximum amount of SCFAs, suggesting that it was potentially a keystone bacterium responsible for PG utilization. PG was degraded into a series of oligosaccharides by *B. xylanisolvens*. In addition, *B. xylanisolvens* was equipped with a large number of divergent CAZymes. Moreover, genomic annotation identified two enzymes, PL17_2 and PL6_1, in *B. xylanisolvens* that are potentially responsible for PG degradation. Our study provides novel insights into PG utilization by the human gut *Bacteroides* species, which has important implications for the development of carbohydrate-based drugs from marine resources.

## Data Availability

The data are available on request from the corresponding authors.

## Supplementary Materials

The following supporting information can be downloaded at https://www.mdpi.com/article/10.3390/md23090348/s1, Figure S1: Analysis of the degradation products of PG by using MS. The oligosaccharides were produced during fermentation of PG by *B. eggerthii*. Assignment of the found ions (A). Chemical structures of the PG oligosaccharides (B); Figure S2: Analysis of the degradation products of PG by using MS. The oligosaccharides were produced during fermentation of PG by *B. finegoldii*. Assignment of the found ions (A). Chemical structures of the PG oligosaccharides (B); Figure S3: Analysis of the degradation products of PG by using MS. The oligosaccharides were produced during fermentation of PG by *B. zhangwenhongii*. Assignment of the found ions (A). Chemical structures of the PG oligosaccharides (B); Table S1: The16S rRNA sequences and alignments used for phylogenetic analysis.

## Abbreviations

The following abbreviations are used in this manuscript:

PG	Polyguluronate
OD	Optical density
TLC	Thin-layer chromatography
SCFAs	Short-chain fatty acids
MS	Mass spectrometry
KEGG	Kyoto Encyclopedia of Genes and Genomes
CAZymes	Carbohydrate active enzymes
PLs	Polysaccharide lyases
GHs	Glycoside hydrolases
AAs	Auxiliary activities
GTs	Glycosyltransferases
CBMs	Carbohydrate-binding modules
CEs	Carbohydrate esterases
Sus	Starch utilization system
PM	Polymannuronate
HPLC	High-performance liquid chromatography
PULs	Polysaccharide utilization loci
