# Supplementary material for: Selective Utilization of Polyguluronate by the Human Gut Bacteroides Species
Source: Mar Drugs. 2025 Aug 29;23(9):348. doi: 10.3390/md23090348 (PMC12471419; doi:10.3390/md23090348)
Supplement: Supplementary file 1 [file marinedrugs-23-00348-s001.zip › marinedrugs-3827248-supplementary.pdf]

Supplementary Figures

A

Fraction	Found Ions (Charge)	Calculated Molar Mass (H Form)	Assignment	
			DP	Sequences
dp2	369.07 (-1)	370.07	2	G-G
udp3	527.09 (-1)	528.09	3	ΔG-G-G
dp3	545.10 (-1)	546.10	3	G-G-G
udp4	351.06 (-2)	704.12	4	ΔG-G-G-G
udp4	703.12 (-1)	704.12	4	ΔG-G-G-G
dp4	721.13 (-1)	722.13	4	G-G-G-G

B

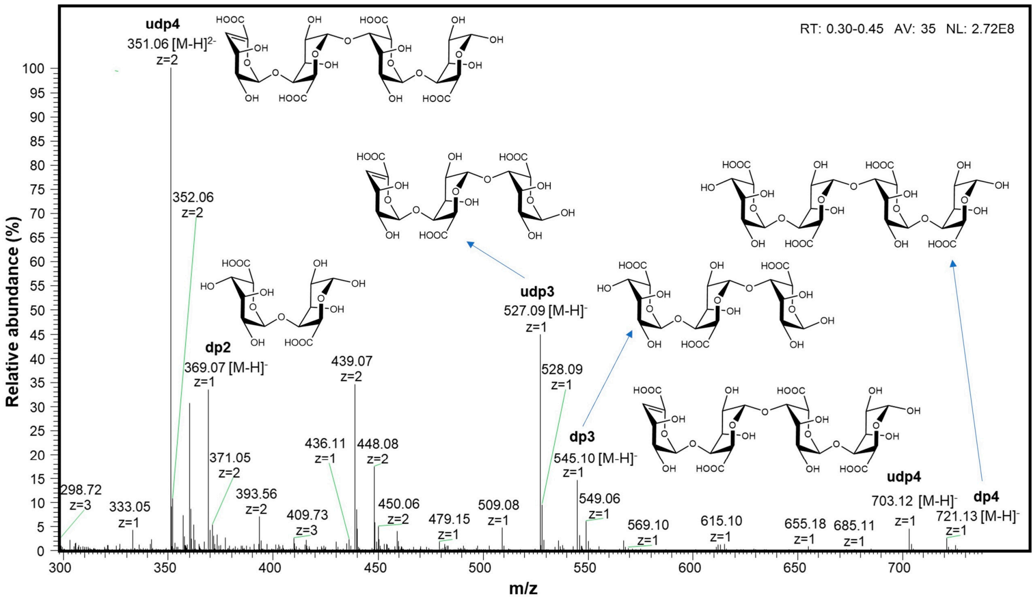

Figure S1. Analysis of the degradation products of PG by using MS. The oligosaccharides were produced during fermentation of PG by *B. eggerthii*. Assignment of the found ions (A). Chemical structures of the PG oligosaccharides (B).

A

Fraction	Found Ions (Charge)	Calculated Molar Mass (H Form)	Assignment	
			DP	Sequences
dp2	369.07 (-1)	370.07	2	G-G
udp3	527.09 (-1)	528.09	3	ΔG-G-G
dp3	545.10 (-1)	546.10	3	G-G-G
udp4	351.06 (-2)	704.12	4	ΔG-G-G-G
udp4	703.12 (-1)	704.12	4	ΔG-G-G-G
dp4	721.13 (-1)	722.13	4	G-G-G-G

B

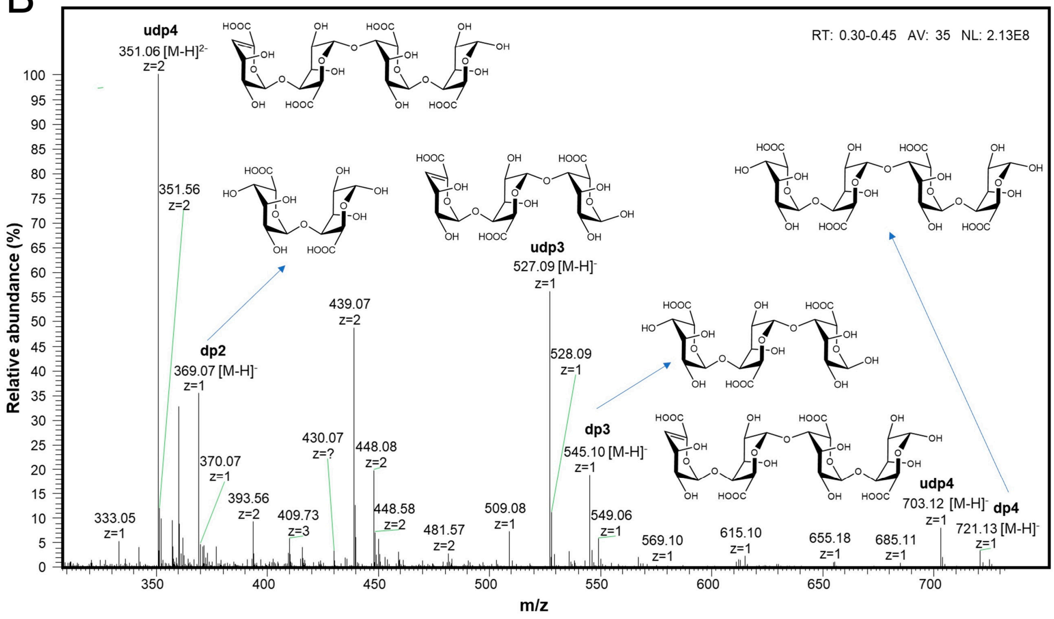

Figure S2. Analysis of the degradation products of PG by using MS. The oligosaccharides were produced during fermentation of PG by *B. finegoldii*. Assignment of the found ions (A). Chemical structures of the PG oligosaccharides (B).

A

Fraction	Found Ions (Charge)	Calculated Molar Mass (H Form)	Assignment	
			DP	Sequences
dp2	369.07 (-1)	370.07	2	G-G
udp3	527.09 (-1)	528.09	3	ΔG-G-G
dp3	545.10 (-1)	546.10	3	G-G-G
udp4	351.06 (-2)	704.12	4	ΔG-G-G-G
udp4	703.12 (-1)	704.12	4	ΔG-G-G-G
dp4	721.13 (-1)	722.13	4	G-G-G-G

B

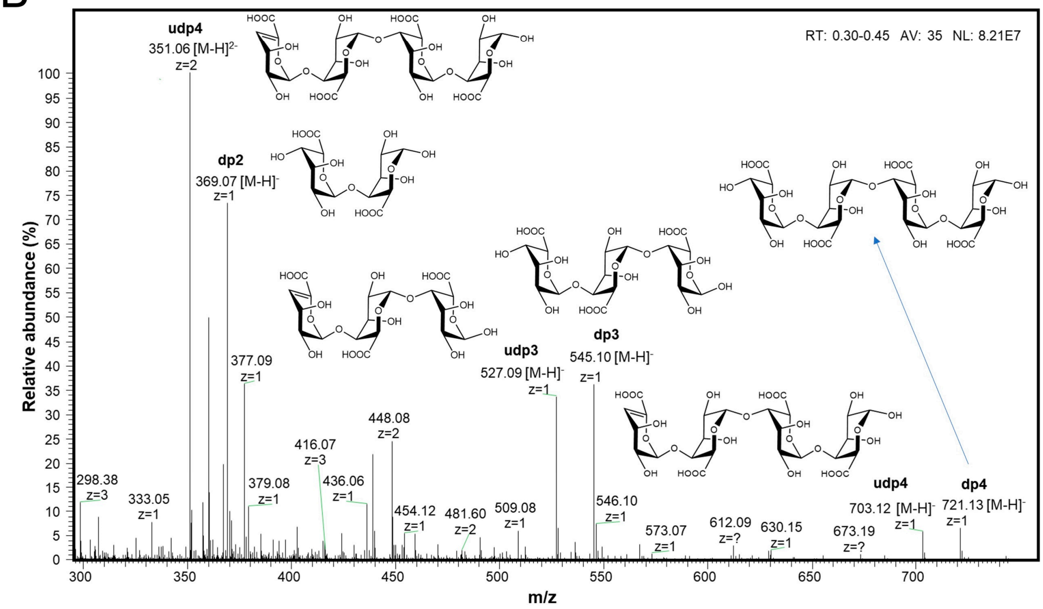

Figure S3. Analysis of the degradation products of PG by using MS. The oligosaccharides were produced during fermentation of PG by *B. zhangwenhongii*. Assignment of the found ions (A). Chemical structures of the PG oligosaccharides (B).

Supplementary Table

Table S1 The16S rRNA sequences and alignments used for phylogenetic analysis.

>*Bacteroides caccae* P2-20
TGGCGACCGGCGCACGGGTGAGTAACACGTATCCAACCTGCCTCATACTCGGGGATAG
CCTTTCGAAAGAAAGATTAATATCCGATAGCATATATTTCCCGCATGGGTTTTATATTAAA
GAAATTCGGTATGAGATGGGGATGCGTTCCATTAGTTTGTTGGCGGGGTAACGGCCCAC
CAAGACTACGATGGATAGGGGTTCTGAGAGGAAGGTCCCCCACATTGGAACTGAGAC
ACGGTCCAAACTCCTACGGGAGGCAGCAGTGAGGAATATTGGTCAATGGACGCGAGT
CTGAACCAGCCAAGTAGCGTGAAGGATGACTGCCCTATGGGTTGTAAACTTCTTTTATA
TGGGAATAAAGTGGTCCACGTGTGGATTTTTGTATGTACCATATGAATAAGGATCGGCT
AACTCCGTGCCAGCAGCCGCGGTAATACGGAGGATCCGAGCGTTATCCGGATTTATTGG
GTTTAAAGGGAGCGTAGGCGGATTGTTAAGTCAGTTGTGAAAGTTTGCGGCTCAACCG
TAAAATTGCAGTTGATACTGGCAGTCTTGAGTGCAGTAGAGGTGGGCGGAATTCGTGG
TGTAGCGGTGAAAT

>*Bacteroides cellulosilyticus* B35-16
ACCGGCGCACGGGTGAGTAACACGTATCCAACCTACCGGTTATTCTGGGATAGCCTTTC
GAAAGAAAGATTAATACCGGATAGTATAACGAGAAGGCATCTTTTTGTTATTAAAGAAT
TTCGATAACCGATGGGGATGCGTTCCATTAGTTTGTTGGCGGGGTAACGGCCCACCAAG
ACATCGATGGATAGGGGTTCTGAGAGGAAGGTCCCCCACATTGGAACTGAGACACGGT
CCAAACTCCTACGGGAGGCAGCAGTGAGGAATATTGGTCAATGGACGAGAGTCTGAA
CCAGCCAAGTAGCGTGAAGGATGACTGCCCTATGGGTTGTAAACTTCTTTTATATGGGA
ATAAAGTGAGCCACGTGTGGCTTTTTGTATGTACCATACGAATAAGGATCGGCTAACTC
CGTGCCAGCAGCCGCGGTAATACGGAGGATCCGAGCGTTATCCGGATTTATTGGGTTTA
AAGGGAGCGTAGGCGGACTATTAAGTCAGCTGTGAAAGTTTGCGGCTCAACCGTAAA
ATTGCAGTTGATACTGGTCGTCTTGAGTGCAGTAGAGGTAGGCGGAATTCGTGGTGTA
GCGGTGAAATGCTTA

>*Bacteroides eggerthii* B21-17
CTTCAATCGATGGCGACCGGCGCACGGGTGAGTAACACGTATCCAACCTGCCGATAAC
TCGGGGATAGCCTTTCGAAAGAAAGATTAATACCCGATAGTATAGTATTTCCGCATGGTT
TCACTATTAAAGAATTTCGGTTATCGATGGGGATGCGTTCCATTAGATAGTTGGCGGGGT
AACGGCCCACCAAGTCAACGATGGATAGGGGTTCTGAGAGGAAGGTCCCCCACATTG
GAACTGAGACACGGTCCAAACTCCTACGGGAGGCAGCAGTGAGGAATATTGGTCAAT
GGACGAGAGTCTGAACCAGCCAAGTAGCGTGAAGGATGACTGCCCTATGGGTTGTAA
ACTTCTTTTATACGGGAATAAAGTGGAGTATGCATACTCCTTTGTATGTACCGTATGAAT
AAGGATCGGCTAACTCCGTGCCAGCAGCCGCGGTAATACGGAGGATCCGAGCGTTATC
CGGATTTATTGGGTTTAAAGGGAGCGTAGGCGGGTGCTTAAGTCAGTTGTGAAAGTTT
GCGGCTCAACCGTAAAATTGCAGTTGATACTGGGCGCCTTGAGTGCAGCATAGGTAGG
CGGAATTCGTGGTGTA

>*Bacteroides faecis* P3-11
AAACTGGAGATGGCGACCGGCGCACGGGTGAGTAACACGTATCCAACCTGCCGATAAC
TCGGGGATAGCCTTTCGAAAGAAAGATTAATACCCGATGGCATAATAGAACCGCATGGT
TTGATTATTAAAGAATTTCGGTTATCGATGGGGATGCGTTCCATTAGGCAGTTGGTGGG
GTAACGGCCCACCAAACCTTCGATGGATAGGGGTTCTGAGAGGAAGGTCCCCCACATT

GGAACTGAGACACGGTCCAAACTCCTACGGGAGGCAGCAGTGAGGAATATTGGTCAA
TGGACGAGAGTCTGAACCAGCCAAGTAGCGTGAAGGATGACTGCCCTATGGGTTGTAA
ACTTCTTTTATATGGGAATAAAGTGGTCCACGTGTGGATTTTTGTATGTACCATATGAATA
AGGATCGGCTAACTCCGTGCCAGCAGCCGCGGTAATACGGAGGATCCGAGCGTTATCC
GGATTTATTGGGTTTAAAGGGAGCGTAGGTGGACAGTTAAGTCAGTTGTGAAAGTTTG
CGGCTCAACCGTAAAATTGCAGTTGATACTGGCTGTCTTGAGTACAGTAGAGGTGGGC
GGAATTCGTGGTGTA
>*Bacteroides finegoldii* B36-12
TGGCGACCGGCGCACGGGTGAGTAACACGTATCCAACCTGCCGATAACTCTGGGATAG
CCTTTCGAAAGAAAGATTAATACCGGATGGCATAGGATTATCGCATGATAATCCTATTAA
AGAATTTCGGTTATCGATGGGGATGCGTTCCATTAGGCAGTTGGTGAGGTAACGGCTCA
CCAAACCTTCGATGGATAGGGGTTCTGAGAGGAAGGTCCCCCACATTGGAACTGAGAC
ACGGTCCAAACTCCTACGGGAGGCAGCAGTGAGGAATATTGGTCAATGGACGGGAGT
CTGAACCAGCCAAGTAGCGTGAAGGATGACTGCCCTATGGGTTGTAAACTTCTTTTATA
CGGGAATAAAGTGGTCCACGTGTGGATTTTTGTATGTACCGTATGAATAAGGATCGGCT
AACTCCGTGCCAGCAGCCGCGGTAATACGGAGGATCCGAGCGTTATCCGGATTTATTGG
GTTTAAAGGGAGCGTAGGTGGATTGTTAAGTCAGTTGTGAAAGTTTGCGGCTCAACCG
TAAAATTGCAGTTGATACTGGCAGTCTTGAGTACAGTAGAGGTGGGCGGAATTCGTGG
TGTAGCGGTGAAAT
>*Bacteroides fragilis* P21-23
AAAGCTTGCTTTCTTTGCTGGCGACCGGCGCACGGGTGAGTAACACGTATCCAACCTG
CCCTTTACTCGGGGATAGCCTTTCGAAAGAAAGATTAATACCCGATAGCATAATGATTCC
GCATGGTTTCATTATTAAAGGATTCCGGTAAAGGATGGGGATGCGTTCCATTAGGTTGTT
GGTGAGGTAACGGCCCACCAAGCCTTCGATGGATAGGGGTTCTGAGAGGAAGGTCCC
CCACATTGGAACTGAGACACGGTCCAAACTCCTACGGGAGGCAGCAGTGAGGAATATT
GGTCAATGGGCGTTAGCCTGAACCAGCCAAGTAGCGTGAAGGATGAAGGCTCTATGGG
TCGTAAACTTCTTTTATATAAGAATAAAGTGCAGTATGTATACTGTTTTGTATGTATTATAT
GAATAAGGATCGGCTAACTCCGTGCCAGCAGCCGCGGTAATACGGAGGATCCGAGCGT
TATCCGGATTTATTGGGTTTAAAGGGAGCGTAGGTGGACTGGTAAGTCAGTTGTGAAA
GTTTGCGGCTCAACCGTAAAATTGCAGTTGATACTGTCAGTCTTGAGTACAGTAGAGGT
GGGCGGAATTC
>*Bacteroides intestinalis* E13-17
CCGGCGCACGGGTGAGTAACACGTATCCAACCTGCCGATTATTCCGGGATAGCCTTTCG
AAAGAAAGATTAATACTGGATAGCATAACGAGAAGGCATCTTCTTGTTATTAAAGAATT
TCGATAATCGATGGGGATGCGTTCCATTAGTTTGTTGGCGGGGTAACGGCCCACCAAGA
CATCGATGGATAGGGGTTCTGAGAGGAAGGTCCCCCACATTGGAACTGAGACACGGTC
CAAACTCCTACGGGAGGCAGCAGTGAGGAATATTGGTCAATGGACGAGAGTCTGAAC
CAGCCAAGTAGCGTGAAGGATGACTGCCCTATGGGTTGTAAACTTCTTTTATATGGGAA
TAAAGTGAGCCACGTGTGGCTTTTTGTATGTACCATACGAATAAGGATCGGCTAACTCC
GTGCCAGCAGCCGCGGTAATACGGAGGATCCGAGCGTTATCCGGATTTATTGGGTTTAA
AGGGAGCGTAGGCGGATTATTAAGTCAGTTGTGAAAGTTTGCGGCTCAACCGTAAAAT
TGCAGTTGATACTGGTAGTCTTGAGTGCAGCAGAGGTAGGCGGAATTCGTGGTGTAGC
GGTGAAATGCTTAG
>*Bacteroides ovatus* B8-7

TGGCGACCGGCGCACGGGTGAGTAACACGTATCCAACCTGCCGATAACTCCGGGATAG
CCTTTCGAAAGAAAGATTAATACCGGATAGCATACGAATATCGCATGATATTTTTATTAA
AGAATTTCGGTTATCGATGGGGATGCGTTCCATTAGTTTGTTGGCGGGGTAACGGCCCA
CCAAGACTACGATGGATAGGGGTTCTGAGAGGAAGGTCCCCCACATTGGAACTGAGA
CACGGTCCAAACTCCTACGGGAGGCAGCAGTGAGGAATATTGGTCAATGGGCGCGAG
CCTGAACCAGCCAAGTAGCGTGAAGGATGAAGGCTCTATGGGTCGTAAACTTCTTTTAT
ATGGGAATAAAGTTTTCCACGTGTGGAATTTTGTATGTACCATATGAATAAGGATCGGCT
AACTCCGTGCCAGCAGCCGCGGTAATACGGAGGATCCGAGCGTTATCCGGATTTATTGG
GTTTAAAGGGAGCGTAGGTGGATTGTTAAGTCAGTTGTGAAAGTTTGCGGCTCAACCG
TAAAATTGCAGTTGAAACTGGCAGTCTTGAGTACAGTAGAGGTGGGCGGAATTCGTGG
TGTAGCGGTGAAAT

>*Bacteroides salyersiae* CSP6

ACCGGCGCACGGGTGAGTAACACGTATCCAACCTGCCCTTTACTCGGGGATAGCCTTT
CGAAAGAAAGATTAATACCCGATGGTATAACATGACCTCCTGGTTTTGTTATTAAAGAA
TTTCGGTAGAGGATGGGGATGCGTTCCATTAGGCAGTTGGCGGGGTAACGGCCCACCA
AACCTTCGATGGATAGGGGTTCTGAGAGGAAGGTCCCCCACATTGGAACTGAGACACG
GTCCAAACTCCTACGGGAGGCAGCAGTGAGGAATATTGGTCAATGGGCGAGAGCCTG
AACCAGCCAAGTAGCGTGAAGGATGACCGCCCTATGGGTTGTAAACTTCTTTTATATGG
GAATAAAGGGTGCCACGTGTGGCATTTTGTATGTACCATATGAATAAGGATCGGCTAAC
TCCGTGCCAGCAGCCGCGGTAATACGGAGGATCCGAGCGTTATCCGGATTTATTGGGTT
TAAAGGGAGCGTAGGTGGACATGTAAGTCAGTTGTGAAAGTTTGCGGCTCAACCGTAA
AATTGCAGTTGAAACTGCGTGTCTTGAGTACAGTAGAGGTGGGCGGAATTCGTGGTGT
AGCGGTGAAATGCTTA

>*Bacteroides stercorirosoris* B32-26

GACCGGCGCACGGGTGAGTAACACGTATCCAACCTGCCGATTATTCCGGGATAGCCTTT
CGAAAGAAAGATTAATACTGGATAGCATAACGAGAAGGCATCTTCTTGTTATTAAAGAA
TTTCGATAATCGATGGGGATGCGTTCCATTAGTTTGTTGGCGGGGTAACGGCTCACCAA
GACATCGATGGATAGGGGTTCTGAGAGGAAGGTCCCCCACATTGGAACTGAGACACG
GTCCAAACTCCTACGGGAGGCAGCAGTGAGGAATATTGGTCAATGGGCGAGAGCCTG
AACCAGCCAAGTAGCGTGAAGGATGACTGCCCTATGGGTTGTAAACTTCTTTTATATGG
GAATAAAGTGCAGTATGTATACTGTTTTGTATGTACCATACGAATAAGGATCGGCTAACT
CCGTGCCAGCAGCCGCGGTAATACGGAGGATCCGAGCGTTATCCGGATTTATTGGGTTT
AAAGGGAGCGTAGGCGGATTATTAAGTCAGTTGTGAAAGTTTGCGGCTCAACCGTAAA
ATTGCAGTTGATACTGGTAGTCTTGAGTGCAGCAGAGGTAGGCGGAATTCGTGGTGTA
GCGGTGAAATGCTT

>*Bacteroides stercoris* P22-28

TTTGATGGATGGCGACCGGCGCACGGGTGAGTAACACGTATCCAACCTGCCGACAACT
CTGGGATAGCCTTTCGAAAGAAAGATTAATACCGGATGGCATAGTTTTCCCGCATGGGA
TGATTATTAAAGAATTTCGGTTGTCGATGGGGATGCGTTCCATTAGGCAGTTGGCGGGG
TAACGGCCCACCAAACCAACGATGGATAGGGGTTCTGAGAGGAAGGTCCCCCACATTG
GAACTGAGACACGGTCCAAACTCCTACGGGAGGCAGCAGTGAGGAATATTGGTCAAT
GGACGAGAGTCTGAACCAGCCAAGTAGCGTGAAGGATGACTGCCCTATGGGTTGTAA
ACTTCTTTTATACGGGAATAAAGTTAGCCACGTGTGGTTTTTTGTATGTACCGTATGAAT
AAGGATCGGCTAACTCCGTGCCAGCAGCCGCGGTAATACGGAGGATCCGAGCGTTATC

CGGATTTATTGGGTTTAAAGGGAGCGTAGGCGGGTTGTTAAGTCAGTTGTGAAAGTTT
GCGGCTCAACCGTAAAATTGCAGTTGATACTGGCGACCTTGAGTGCAACAGAGGTAGG
CGGAATTCGTGGTGTAG

>*Bacteroides thetaiotaomicron* E1-7

AAACTGGAGATGGCGACCGGCGCACGGGTGAGTAACACGTATCCAACCTGCCGATAAC
TCGGGGATAGCCTTTCGAAAGAAAGATTAATACCCGATGGTATAATTAGACCGCATGGT
CTTGTTATTAAAGAATTTCGGTTATCGATGGGGATGCGTTCCATTAGGCAGTTGGTGAG
GTAACGGCTCACCAAACCTTCGATGGATAGGGGTTCTGAGAGGAAGGTCCCCCACATT
GGAACTGAGACACGGTCCAAACTCCTACGGGAGGCAGCAGTGAGGAATATTGGTCAA
TGGGCGCAGGCCTGAACCAGCCAAGTAGCGTGAAGGATGACTGCCCTATGGGTTGTAA
ACTTCTTTTATATGGGAATAAAGTTTTCCACGTGTGGAATTTTGTATGTACCATATGAATA
AGGATCGGCTAACTCCGTGCCAGCAGCCGCGGTAATACGGAGGATCCGAGCGTTATCC
GGATTTATTGGGTTTAAAGGGAGCGTAGGTGGACAGTTAAGTCAGTTGTGAAAGTTTG
CGGCTCAACCGTAAAATTGCAGTTGATACTGGCTGTCTTGAGTACAGTAGAGGTGGGC
GGAATTCGTGGTGTA

>*Bacteroides uniformis* P30-16

TAAGTTTGATGGCGACCGGCGCACGGGTGAGTAACACGTATCCAACCTGCCGATGACT
CGGGGATAGCCTTTCGAAAGAAAGATTAATACCCGATGGCATAGTTCTTCCGCATGGTG
GAACTATTAAAGAATTTCGGTCATCGATGGGGATGCGTTCCATTAGGTTGTTGGCGGGG
TAACGGCCCACCAAGCCTTCGATGGATAGGGGTTCTGAGAGGAAGGTCCCCCACATTG
GAACTGAGACACGGTCCAAACTCCTACGGGAGGCAGCAGTGAGGAATATTGGTCAAT
GGACGAGAGTCTGAACCAGCCAAGTAGCGTGAAGGATGACTGCCCTATGGGTTGTAA
ACTTCTTTTATACGGGAATAAAGTGAGGCACGTGTGCCTTTTTGTATGTACCGTATGAAT
AAGGATCGGCTAACTCCGTGCCAGCAGCCGCGGTAATACGGAGGATCCGAGCGTTATC
CGGATTTATTGGGTTTAAAGGGAGCGTAGGCGGACGCTTAAGTCAGTTGTGAAAGTTT
GCGGCTCAACCGTAAAATTGCAGTTGATACTGGGTGTCTTGAGTACAGTAGAGGCAGG
CGGAATTCGTGGTGTAG

>*Bacteroides xylanisolvens* AY11-1

GCAAACTAAAGATGGCGACCGGCGCACGGGTGAGTAACACGTATCCAACCTGCCGATA
ACTCGGGGATAGCCTTTCGAAAGAAAGATTAATATCCGATAGTATATTAAAACCGCATG
GTTTTACTATTAAAGAATTTCGGTTATCGATGGGGATGCGTTCCATTAGTTTGTTGGCGG
GGTAACGGCCCACCAAGACTACGATGGATAGGGGTTCTGAGAGGAAGGTCCCCCACAT
TGGAACTGAGACACGGTCCAAACTCCTACGGGAGGCAGCAGTGAGGAATATTGGTCA
ATGGACGAGAGTCTGAACCAGCCAAGTAGCGTGAAGGATGACTGCCCTATGGGTTGTA
AACTTCTTTTATATGGGAATAAAGTATTCCACGTGTGGAATTTTGTATGTACCATATGAAT
AAGGATCGGCTAACTCCGTGCCAGCAGCCGCGGTAATACGGAGGATCCGAGCGTTATC
CGGATTTATTGGGTTTAAAGGGAGCGTAGGTGGATTGTTAAGTCAGTTGTGAAAGTTTG
CGGCTCAACCGTAAAATTGCAGTTGAAACTGGCAGTCTTGAGTACAGTAGAGGTGGGC
GGAATTCGTGGTG

>*Bacteroides zhangwenhongii* 10-10

CTTGCAAACTGAAGATGGCGACCGGCGCACGGGTGAGTAACACGTATCCAACCTGCC
GATAACTCGGGGATAGCCTTTCGAAAGAAAGATTAATACCGGATGGCATATGATTATCG
CATGATAATCCTATTAAAGAATTTCGGTTATCGATGGGGATGCGTTCCATTAGTTTGTTG
GCGGGGTAACGGCCCACCAAGACCGCGATGGATAGGGGTTCTGAGAGGAAGGTCCCC

CACATTGGAACTGAGACACGGTCCAAACTCCTACGGGAGGCAGCAGTGAGGAATATT
GGTCAATGGACGAGAGTCTGAACCAGCCAAGTAGCGTGAAGGATGACTGCCCTATGG
GTTGTAAACTTCTTTTATACGGGAATAAAGTGGTCCACGTGTGGATTTTTGTATGTACCG
TATGAATAAGGATCGGCTAACTCCGTGCCAGCAGCCGCGGTAATACGGAGGATCCGAG
CGTTATCCGGATTTATTGGGTTTAAAGGGAGCGTAGGTGGACAGTTAAGTCAGTTGTGA
AAGTTTGCGGCTCAACCGTAAAATTGCAGTTGATACTGGCTGTCTTGAGTACAGTAGA
GGTGGGCGGAATTCGTG

>*Bacteroides parvus* S4-M12

TGCTTTCTTTGCTGGCGACCGGCGCACGGGTGAGTAACACGTATCCAACCTGCCGATG
ACTCGGGGATAGCCTTTCGAAAGAAAGATTAATACCCGATGGTATATCTGAAAGGCATC
TTTCAGCTATTAAAGAATTTCGGTCATTGATGGGGATGCGTTCCATTAGGTTGTTGGCGG
GGTAACGGCCCACCAAGCCATCGATGGATAGGGGTTCTGAGAGGAAGGTCCCCCACAT
TGGAACTGAGACACGGTCCAAACTCCTACGGGAGGCAGCAGTGAGGAATATTGGTCA
ATGGACGAGAGTCTGAACCAGCCAAGTAGCGTGAAGGATGACTGCCCTATGGGTTGTA
AACTTCTTTTATACGGGAATAAAGTTAGGCACGTGTGCCTTTTTGTATGTACCGTATGAA
TAAGGATCGGCTAACTCCGTGCCAGCAGCCGCGGTAATACGGAGGATCCGAGCGTTAT
CCGGATTTATTGGGTTTAAAGGGAGCGTAGGCGGATGCTTAAGTCAGTTGTGAAAGTTT
GCGGCTCAACCGTAAAATTGCAGTTGATACTGGGTGTCTTGAGTACAGTAGAGGCAGG
CGGAATTCGTGGTG

>*Bacteroides multiformis* ZF-8

GTGAGTAACACGTATCCAACCTGCCGATTATTCCGGGATAGCCTTTCGAAAGAAAGATT
AATACTGGATAGTATAACGAGAAGGCATCTTTTTGTTATTAAAGAATTTCGATAATCGAT
GGGGATGCGTTCCATTAGCTTGTTGGCGGGGTAACGGCCCACCAAGGCATCGATGGAT
AGGGGTTCTGAGAGGAAGGTCCCCCACATTGGAACTGAGACACGGTCCAAACTCCTA
CGGGAGGCAGCAGTGAGGAATATTGGTCAATGGACGAGAGTCTGAACCAGCCAAGTA
GCGTGAAGGATGACTGCCCTATGGGTTGTAAACTTCTTTTATATGGGAATAAAGTGAGC
CACGTGTGGCTTTTTGTATGTACCATACGAATAAGGATCGGCTAACTCCGTGCCAGCAG
CCGCGGTAATACGGAGGATCCAAGCGTTATCCGGATTTATTGGGTTTAAAGGGAGCGTA
GGCGGACTATTAAGTCAGCTGTGAAAGTTTGCGGCTCAACCGTAAAATTGCAGTTGAT
ACTGGTCGTCTTGAGTGCAGTAGAGGTAGGCGGAATTCGTGGTGTAGCGGTGAAATGC
TTAGATATCACGAAG
